# Molecular Analysis of Linezolid-Resistant Clinical Isolates of *Mycobacterium abscessus*

**DOI:** 10.1128/AAC.01842-18

**Published:** 2019-01-29

**Authors:** Meiping Ye, Liyun Xu, Yuzhen Zou, Bing Li, Qi Guo, Yongjie Zhang, Mengling Zhan, Benyong Xu, Fangyou Yu, Zhemin Zhang, Haiqing Chu

**Affiliations:** a Department of Respiratory Medicine, Shanghai Pulmonary Hospital, Tongji University School of Medicine, Shanghai, China; b Tongji University School of Medicine, Shanghai, China; c Department of Clinical Laboratory Medicine, Shanghai Pulmonary Hospital, Tongji University School of Medicine, Shanghai, China; d Shanghai Key Laboratory of Tuberculosis, Shanghai Pulmonary Hospital, Tongji University School of Medicine, Shanghai, China

**Keywords:** linezolid, *Mycobacterium abscessus*, drug resistance mechanisms

## Abstract

A total of 194 Mycobacterium abscessus isolates were collected from patients, and the whole genomes were sequenced. Eighty-five (43.8%) isolates showed linezolid (LZD) resistance. Only 8.2% of resistant isolates harbored 23S rRNA mutations. Quantitative real-time PCR (qRT-PCR) revealed higher transcriptional levels of efflux pumps *lmrS* and *mmpL9* in LZD-resistant isolates. Genome comparative analysis identified several new LZD resistance-associated genes. This study highlights the role of efflux pumps in LZD-resistant M. abscessus and proposes potential target genes for further studies.

## Abstract (precis)

A total of 194 Mycobacterium abscessus isolates were collected from patients, and the whole genomes were sequenced. Eighty-five (43.8%) isolates showed linezolid (LZD) resistance.

## INTRODUCTION

Mycobacterium abscessus, one of the important nontuberculous mycobacterial (NTM) pathogens (1), causes human infections with high morbidity and mortality (2, 3). However, chemotherapeutic options against infections caused by M. abscessus are very limited due to its innate resistance to multiple antibiotic classes (4).

Linezolid (LZD), the first member of the oxazolidinone class, has been reported to be one of the most potent antibiotics against infections caused by M. abscessus (3, 5). Unfortunately, LZD-resistant M. abscessus strains are emerging worldwide (6, 7). Almost all resistance mechanisms against LZD reported to date involve alterations of LZD binding sites, including mutations in 23S rRNA and ribosomal proteins (L3, L4, and L22), or modifications of 23S rRNA, which were mainly investigated in M. tuberculosis, Staphylococcus spp., and Enterococcus spp. (8–10).

To date, knowledge on LZD resistance mechanisms in M. abscessus is limited. In this study, we collected 194 M. abscessus clinical isolates and sequenced all the genomes. Further investigation of resistance mechanism was performed in 85 LZD-resistant clinical isolates.

### Screening of LZD-resistant isolates.

One hundred ninety-four M. abscessus isolates were collected in Shanghai Pulmonary Hospital from sputum and bronchoalveolar lavage fluid samples between January 2012 and December 2017. LZD MICs were determined by a broth microdilution method according to CLSI guidelines, and the breakpoints were interpreted according to CLSI document M24-A2 (≤8 mg/liter, susceptible; 16 mg/liter, intermediate resistant; ≥32 mg/liter, fully resistant) (11). Mycobacterium peregrinum ATCC 700686 and Staphylococcus aureus ATCC 29213 served as the control reference strains.

**FIG 1:**
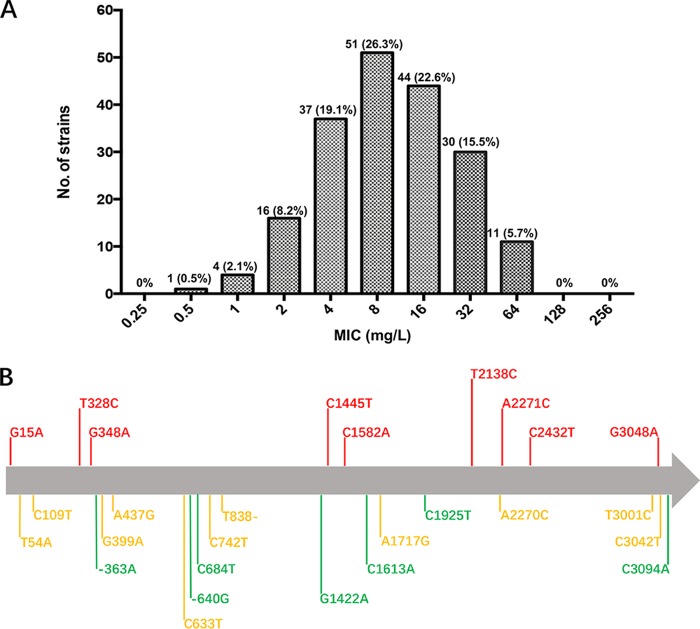
(A) Distribution of LZD MICs of 194 *M. abscessus* clinical isolates. The number and proportion of isolates are labeled on the top of each bar. (B) Schematic diagram of mutations in the 23S rRNA among 194 *M. abscessus* clinical isolates. Green indicates that the mutation is present only in LZD-susceptible isolates, red indicates that the mutation is present only in LZD-resistant isolates, and yellow indicates that the mutation is present in both LZD-susceptible and -resistant isolates.

The MICs of LZD against 194 M. abscessus isolates ranged from 0.5 to 64 mg/liter, with an MIC_50_ of 8 mg/liter and an MIC_90_ of 32 mg/liter (Fig. 1A). Eighty-five (43.8%) isolates were resistant to LZD, 44 (22.6%) of which were intermediate resistant and 41 (21.2%) which were fully resistant. The remaining 109 (56.2%) isolates were susceptible to LZD. The LZD resistance rate of M. abscessus was high (43.8%), which is consistent with findings from previous studies (6, 7, 12–16).

### Alternations in the LZD target sites.

Whole genomes of the 194 strains were sequenced (BioProject PRJNA488058 from this study and PRJNA448987 and PRJNA448987 from our previous studies), including 96 isolated in 2017 and 98 isolated during 2012 to 2016 (13, 17). The sequences of the entire 23S rRNA, L3, L4, and L22 proteins were extracted from the whole-genome sequence data of each strain and compared with those from reference strain ATCC 19977. A total of 26 mutation types were observed in 23S rRNA. Detailed information about the mutations is listed in Table S1 in the supplemental material. Nine mutations were found in 7 (8.2%) LZD-resistant strains, indicating that these mutations contributed to LZD resistance (Fig. 1B, red). Other 17 mutations in 23S rRNA were present in either susceptible strains or in both susceptible and resistant strains, suggesting that they do not contribute to LZD resistance. No meaningful mutations were found in L3, L4, and L22 in LZD-resistant strains. These results suggest that a mutation in ribosomal proteins is not responsible for LZD resistance in most of the strains isolated in this study.

The methyltransferase genes *cfr*, *rlmN*, and *spr033* and the pseudouridine synthase gene *rulC* that modify the 23S rRNA at the LZD binding sites are known to affect LZD susceptibility (18–21). However, none of them were found in our 194 isolates.

### Efflux pumps play an important role in LZD resistance of M. abscessus.

Several efflux pumps, including *drrABC, rv0987, lmrS, acrAB, mmpL9, acrF*, and *optrA*, have been reported to extrude LZD (22). Therefore, efflux pump inhibition tests were conducted with a combination of phenylalanine-arginine β-naphthylamide (PaβN, 20 mg/liter), carbonyl cyanide 3-chlorophenylhydrazone (CCCP; 5 mg/liter), and reserpine (12 mg/liter) (23, 24). As shown in Table 1, these inhibitors could decrease MICs of LZD in over 50% of resistant strains, supporting the role of efflux pumps in LZD resistance of M. abscessus.

**TABLE 1:** MIC fold changes of 41 linezolid-resistant *M. abscessus* strains upon addition of efflux pump inhibitors^*a*^

Treatment	No. (%) with MIC fold change decrease of:		
	1	2	4
Linezolid + PAβN	12 (29.3)	26 (63.4)	3 (7.3)
Linezolid + CCCP	10 (24.4)	25 (61.0)	6 (14.6)
Linezolid + reserpine	19 (46.3)	20 (48.8)	2 (4.9)

a 1 represents no MIC fold change.

Sequence alignment showed that the homologs of *drrABC, rv0987, lmrS, acrAB, mmpL9*, and *acrF* were present in all of the 194 M. abscessus isolates, except for *optrA*.

**FIG 2:**
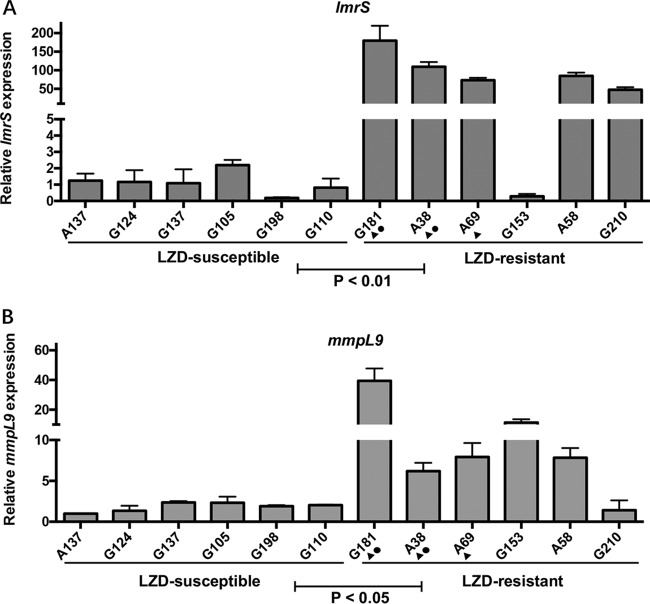
qRT-PCR analysis of transcript levels of *lmrS* (A) and *mmpL9* (B). Error bars represent the standard errors of each data point. A *t* test was used to test the differences among groups. Triangles (▲) indicate the strains whose LZD MIC decreased 4-fold after treatment with the inhibitor PAβN. Circles (●) indicate the strains whose LZD MIC decreased 4-fold after treatment with the inhibitor reserpine.

Therefore, LZD-resistant isolates with a significant MIC fold change (4-fold) upon efflux pump inhibition (*n* = 6), along with 6 randomly selected LZD-susceptible isolates (MICs, 0.5 to 4 mg/liter), were selected and subjected to quantitative real-time PCR (qRT-PCR) analysis, as previously described (17). Primer pairs for amplification of each gene were as follows: *mmpL9*, ACGTCATTTCAGCTCTGCCA/AAGGGGCGGGTGATACTTTG; *drrC*, GTCGAGTACAGCACGCGATA/TAATCCGACCAGCAACCCAC; *drrA*, GTCCCGGATTGGCGAAATTG/GCTGCTTTTCCATCTCGCTG; *lmrS*, TGGTCAATGCTCGCATTCCT/ATCGGGTATCCCCTTGGTCA; *acrF*, ACTTCGTTGCGTTCCTCGAT/AGCGTTGTCACTCAACACCA; and *acrB*, GATTCGGTATCGGTGGCTGT/CCGGATTCTCCTCGACGAAC. As shown in Fig. 2, the LZD-resistant strains had >50-fold (*P* = 0.004) and >5-fold (*P* = 0.04) increased transcriptional levels of *lmrS* and *mmpL9*, respectively, compared to the LZD-susceptible strains. These results indicated that efflux pumps *lmrS* and *mmpL9* play an important role in LZD resistance in M. abscessus. No difference in the transcription levels of *drrABC, rv0987, acrAB*, or *acrF* was observed between the LZD-susceptible and resistant groups (data not shown).

### Whole-genome comparative analysis.

For 25% of the LZD-resistant M. abscessus isolates in this study, resistance could not be explained by known mechanisms, suggesting the presence of novel mechanisms for LZD resistance. Accordingly, genome comparative analysis was conducted and identified 24 genes that were highly associated with LZD resistance (*P* < 0.01), such as genes encoding MmpL10, which is known to mediate drug resistance in M. tuberculosis (25), and FabG, which is required for antibiotic resistance in P. aeruginosa (26). Detailed information for these genes is listed in Table S2.

In conclusion, this study suggests that rather than mutations or modifications of LZD target sites, efflux pumps played a predominant role in LZD resistance of M. abscessus. Whole-genome sequencing and comparative analyses also identified new LZD resistance-associated genes, which set the foundation for elucidation of the mechanism of LZD resistance in M. abscessus.

### Accession number(s).

Whole-genome sequences have been deposited under BioProject no. PRJNA488058.

## Supplementary Material

Supplemental file 1

## References

[1] Kasperbauer SH, De Groote MA 2015 The treatment of rapidly growing mycobacterial infections. Clin Chest Med 36:67–78. doi:10.1016/j.ccm.2014.10.004. 25676520

[2] Floto RA, Olivier KN, Saiman L, Daley CL, Herrmann JL, Nick JA, Noone PG, Bilton D, Corris P, Gibson RL, Hempstead SE, Koetz K, Sabadosa KA, Sermet-Gaudelus I, Smyth AR, van Ingen J, Wallace RJ, Winthrop KL, Marshall BC, Haworth CS 2016 US Cystic Fibrosis Foundation and European Cystic Fibrosis Society consensus recommendations for the management of non-tuberculous mycobacteria in individuals with cystic fibrosis: executive summary. Thorax 71:88–90. doi:10.1136/thoraxjnl-2015-207983. 26678435 PMC4717423

[3] Griffith DE, Aksamit T, Brown-Elliott BA, Catanzaro A, Daley C, Gordin F, Holland SM, Horsburgh R, Huitt G, Iademarco MF, Iseman M, Olivier K, Ruoss S, von Reyn CF, Wallace RJ Jr, Winthrop K, ATS Mycobacterial Diseases Subcommittee, American Thoracic Society, Infectious Disease Society of America. 2007 An official ATS/IDSA statement: diagnosis, treatment, and prevention of nontuberculous mycobacterial diseases. Am J Respir Crit Care Med 175:367–416. doi:10.1164/rccm.200604-571ST. 17277290

[4] Nessar R, Cambau E, Reyrat JM, Murray A, Gicquel B 2012 *Mycobacterium abscessus*: a new antibiotic nightmare. J Antimicrob Chemother 67:810–818. doi:10.1093/jac/dkr578. 22290346

[5] Haworth CS, Banks J, Capstick T, Fisher AJ, Gorsuch T, Laurenson IF, Leitch A, Loebinger MR, Milburn HJ, Nightingale M, Ormerod P, Shingadia D, Smith D, Whitehead N, Wilson R, Floto RA 2017 British Thoracic Society guidelines for the management of non-tuberculous mycobacterial pulmonary disease (NTM-PD). Thorax 72:ii1–ii64. doi:10.1136/thoraxjnl-2017-210927. 29054853

[6] Zhang Z, Lu J, Song Y, Pang Y 2018 *In vitro* activity between linezolid and other antimicrobial agents against *Mycobacterium abscessus* complex. Diagn Microbiol Infect Dis 90:31–34. doi:10.1016/j.diagmicrobio.2017.09.013. 29089153

[7] Lee MC, Sun PL, Wu TL, Wang LH, Yang CH, Chung WH, Kuo AJ, Liu TP, Lu JJ, Chiu CH, Lai HC, Chen NY, Yang JH, Wu TS 2017 Antimicrobial resistance in *Mycobacterium abscessus* complex isolated from patients with skin and soft tissue infections at a tertiary teaching hospital in Taiwan. J Antimicrob Chemother 72:2782–2786. doi:10.1093/jac/dkx212. 29091186

[8] Long KS, Vester B 2012 Resistance to linezolid caused by modifications at its binding site on the ribosome. Antimicrob Agents Chemother 56:603–612. doi:10.1128/AAC.05702-11. 22143525 PMC3264260

[9] Zong Z, Jing W, Shi J, Wen S, Zhang T, Huo F, Shang Y, Liang Q, Huang H, Pang Y 2018 Comparison of *in vitro* activity and MIC distributions between the novel oxazolidinone delpazolid and linezolid against multidrug-resistant and extensively drug-resistant *Mycobacterium tuberculosis* in China. Antimicrob Agents Chemother 62:e00165-18. doi:10.1128/AAC.00165-18. 29844043 PMC6105784

[10] Tian Y, Li T, Zhu Y, Wang B, Zou X, Li M 2014 Mechanisms of linezolid resistance in staphylococci and enterococci isolated from two teaching hospitals in Shanghai, China. BMC Microbiol 14:292. doi:10.1186/s12866-014-0292-5. 25420718 PMC4245736

[11] Clinical and Laboratory Standards Institute. 2011 Susceptibility testing of mycobacteria, nocardiae, and other aerobic actinomycetes; approved standard, 2nd ed CLSI document M24-A2. Clinical and Laboratory Standards Institute, Wayne, PA. 31339680

[12] Hatakeyama S, Ohama Y, Okazaki M, Nukui Y, Moriya K 2017 Antimicrobial susceptibility testing of rapidly growing mycobacteria isolated in Japan. BMC Infect Dis 17:197. doi:10.1186/s12879-017-2298-8. 28270102 PMC5341166

[13] Li B, Yang S, Chu H, Zhang Z, Liu W, Luo L, Ma W, Xu X 2017 Relationship between antibiotic susceptibility and genotype in *Mycobacterium abscessus* clinical isolates. Front Microbiol 8:1739. doi:10.3389/fmicb.2017.01739. 28959242 PMC5603792

[14] Zhang Z, Lu J, Liu M, Wang Y, Zhao Y, Pang Y 2017 *In vitro* activity of clarithromycin in combination with other antimicrobial agents against *Mycobacterium abscessus* and *Mycobacterium massiliense*. Int J Antimicrob Agents 49:383–386. doi:10.1016/j.ijantimicag.2016.12.003. 28188830

[15] Kusuki M, Osawa K, Arikawa K, Tamura M, Shigemura K, Shirakawa T, Nakamura T, Nakamachi Y, Fujisawa M, Saegusa J, Tokimatsu I 2018 Determination of the antimicrobial susceptibility and molecular profile of clarithromycin resistance in the *Mycobacterium abscessus* complex in Japan by variable number tandem repeat analysis. Diagn Microbiol Infect Dis 91:256–259. doi:10.1016/j.diagmicrobio.2018.02.008. 29550059

[16] Cho EH, Huh HJ, Song DJ, Lee SH, Kim CK, Shin SY, Ki C-S, Jhun BW, Moon SM, Kwon OJ, Koh W-J, Lee NY 29 8 2018 Drug susceptibility patterns of *Mycobacterium abscessus* and *Mycobacterium massiliense* isolated from respiratory specimens. Diagn Microbiol Infect Dis doi:10.1016/j.diagmicrobio.2018.08.008. 30236529

[17] Li B, Ye M, Guo Q, Zhang Z, Yang S, Ma W, Yu F, Chu H 2018 Determination of MIC distribution and mechanisms of decreased susceptibility to bedaquiline among clinical isolates of *Mycobacterium abscessus*. Antimicrob Agents Chemother 62:e00175-18. doi:10.1128/AAC.00175-18. 29712658 PMC6021634

[18] Locke JB, Morales G, Hilgers M, G CK, Rahawi S, Jose Picazo J, Shaw KJ, Stein JL 2010 Elevated linezolid resistance in clinical *cfr*-positive *Staphylococcus aureus* isolates is associated with co-occurring mutations in ribosomal protein L3. Antimicrob Agents Chemother 54:5352–5355. doi:10.1128/AAC.00714-10. 20837755 PMC2981277

[19] LaMarre JM, Howden BP, Mankin AS 2011 Inactivation of the indigenous methyltransferase RlmN in *Staphylococcus aureus* increases linezolid resistance. Antimicrob Agents Chemother 55:2989–2991. doi:10.1128/AAC.00183-11. 21444696 PMC3101465

[20] Billal DS, Feng J, Leprohon P, Legare D, Ouellette M 2011 Whole genome analysis of linezolid resistance in *Streptococcus pneumoniae* reveals resistance and compensatory mutations. BMC Genomics 12:512. doi:10.1186/1471-2164-12-512. 22004526 PMC3212830

[21] Conrad J, Sun D, Englund N, Ofengand J 1998 The *rluC* gene of *Escherichia coli* codes for a pseudouridine synthase that is solely responsible for synthesis of pseudouridine at positions 955, 2504, and 2580 in 23S ribosomal RNA. J Biol Chem 273:18562–18566. doi:10.1074/jbc.273.29.18562. 9660827

[22] Srivastava S, Magombedze G, Koeuth T, Sherman C, Pasipanodya JG, Raj P, Wakeland E, Deshpande D, Gumbo T 2017 Linezolid dose that maximizes sterilizing effect while minimizing toxicity and resistance emergence for tuberculosis. Antimicrob Agents Chemother 61:e00751-17. doi:10.1128/AAC.00751-17. 28584143 PMC5527615

[23] Pasca MR, Guglierame P, Arcesi F, Bellinzoni M, De Rossi E, Riccardi G 2004 Rv2686c-Rv2687c-Rv2688c, an ABC fluoroquinolone efflux pump in *Mycobacterium tuberculosis*. Antimicrob Agents Chemother 48:3175. doi:10.1128/AAC.48.8.3175-3178.2004. 15273144 PMC478549

[24] Li XZ, Plesiat P, Nikaido H 2015 The challenge of efflux-mediated antibiotic resistance in Gram-negative bacteria. Clin Microbiol Rev 28:337–418. doi:10.1128/CMR.00117-14. 25788514 PMC4402952

[25] Domenech P, Reed MB, Barry CE III. 2005 Contribution of the *Mycobacterium tuberculosis* MmpL protein family to virulence and drug resistance. Infect Immun 73:3492–3501. doi:10.1128/IAI.73.6.3492-3501.2005. 15908378 PMC1111821

[26] Caughlan RE, Jones AK, Delucia AM, Woods AL, Xie L, Ma B, Barnes SW, Walker JR, Sprague ER, Yang X, Dean CR 2012 Mechanisms decreasing in vitro susceptibility to the LpxC inhibitor CHIR-090 in the Gram-negative pathogen *Pseudomonas aeruginosa*. Antimicrob Agents Chemother 56:17–27. doi:10.1128/AAC.05417-11. 22024823 PMC3256010

